# Thromboelastometry identifies coagulopathy associated with liver failure and disseminated intravascular coagulation caused by yellow fever, guiding specific hemostatic therapy: a case report

**DOI:** 10.5935/0103-507X.20200078

**Published:** 2020

**Authors:** Tomaz Crochemore, Felício Aragão Savioli, João Carlos de Campos Guerra, Erika Maria do Nascimento Kalmar

**Affiliations:** 1 Critical Care Medicine, Hospital Leforte - São Paulo (SP), Brazil.; 2 Hematology and Laboratory Center, Hospital Israelita Albert Einstein - São Paulo (SP), Brazil.; 3 Infectology, Hospital Israelita Albert Einstein - São Paulo (SP), Brazil.

**Keywords:** Yellow fever, Thromboelastography, Fibrinogen, Vitamin K, Disseminated intravascular coagulation, Hemostatics, Thrombocytopenia, Coagulopathy, Febre amarela, Tromboelastometria, Fibrinogênio, Vitamina K, Coagulação intravascular disseminada, Hemostáticos, Trombocitopenia, Coagulopatia

## Abstract

This case report a severe case of yellow fever complicated by liver failure and disseminated intravascular coagulation. Thromboelastometry was capable of identifying clotting disorders and guiding hemostatic therapy. We report the case of a 23-year-old male admitted to the Intensive Care Unit with sudden onset of fever, generalized muscle pain associated with liver failure, and disseminated intravascular coagulation. The results of conventional laboratory tests showed thrombocytopenia, whereas thromboelastometry suggested coagulopathy with slight hypofibrinogenemia, clotting factor consumption, and, consequently, an increased risk of bleeding. Unlike conventional laboratory tests, thromboelastometry identified the specific coagulation disorder and thereby guided hemostatic therapy. Both fibrinogen concentrates and vitamin K were administered, and no blood component transfusion was required, even in the presence of thrombocytopenia. Administration of hemostatic drugs, including fibrinogen concentrate and vitamin K, improved thromboelastometric parameters, correcting the complex coagulation disorder. Blood component transfusion was not performed, and there was no bleeding.

## Abstract (pt)

Este relato de caso detalha um caso grave de febre amarela complicada por insuficiência hepática e coagulação intravascular disseminada. A tromboelastometria foi capaz de identificar os distúrbios da coagulação e orientar o tratamento hemostático. Relatamos o caso de um homem com 23 anos de idade admitido na unidade de terapia intensiva com quadro com início abrupto de febre e dor muscular generalizada associados a insuficiência hepática e coagulação intravascular disseminada. Os resultados dos exames laboratoriais convencionais revelaram trombocitopenia, enquanto a tromboelastometria sugeriu coagulopatia com discreta hipofibrinogenemia, consumo de fatores de coagulação e, consequentemente, aumento do risco de sangramento. Diferentemente dos exames laboratoriais convencionais, a tromboelastometria identificou o distúrbio de coagulação específico e, assim, orientou o tratamento hemostático. Administraram-se concentrados de fibrinogênio e vitamina K, não sendo necessária a transfusão de qualquer componente do sangue, mesmo na presença de trombocitopenia. A tromboelastometria permitiu a identificação precoce da coagulopatia e ajudou a orientar a terapêutica hemostática. A administração de fármacos hemostáticos, incluindo concentrados de fibrinogênio e vitamina K, melhorou os parâmetros tromboelastométricos, com correção do transtorno da coagulação. Não se realizou transfusão de hemocomponentes, e não ocorreu qualquer sangramento.

## INTRODUCTION

Yellow fever is an acute viral hemorrhagic disease transmitted by infected mosquitoes. The “yellow” in the name comes from the jaundice that can be found in some patients with severe disease. Diagnosis depends on travel history to an endemic area, exposure to infected mosquitoes, vaccination history, symptoms, and laboratory findings.^(1,2)^ The virus is endemic in tropical areas of Africa and Central and South America.^(3-5)^ Most cases are subclinical or mildly symptomatic with an excellent prognosis. Of symptomatic patients, 15% will develop severe disease, among which 30% to 50% will die. Despite its relevance, there is a lack of literature about the clinical and pathological features of yellow fever, in part due to its occurrence in remote areas, in which access to sophisticated diagnostic procedures is limited. The virus causing yellow fever is an RNA virus, the prototype of the genus Flavivirus (family Flaviviridae). Currently, it is an important cause of hemorrhagic fever-related death worldwide.^(6)^

Yellow fever is a viral sepsis whose most common clinical manifestations include sudden onset of fever with prostration to more serious illness associated with multiorgan dysfunction, poor outcome and high mortality. Most patients recover after 3 to 6 days, but 15 to 25% enter a more toxic second phase within 24 hours after an initial remission. The intoxication period, featuring the severe form of the disease, comes with high fever, severe abdominal pain, vomiting, and acute fulminant hepatitis, with fast deterioration of liver function and jaundice.^(6)^ Hepatitis-induced coagulopathy may be associated with severe hemorrhagic manifestations, including petechiae, ecchymoses, epistaxis, and hematemesis.^(7)^ Decreased synthesis of clotting factors by the liver and disseminated intravascular coagulation (DIC) are the main causes of bleeding. Both hemorrhage and poor outcome are strongly correlated with high levels of proinflammatory and anti-inflammatory cytokines.^(8)^ Multiorgan dysfunction, including liver, renal and myocardial failure with shock and DIC, may be found.

Disseminated intravascular coagulation is an acquired syndrome characterized by the systemic activation of blood coagulation, which excessively generates intravascular thrombin and fibrin, resulting in the thrombosis of small- to medium-sized vessels and organ dysfunction. The continuous consumption of coagulation factors and platelets associated with activation of the fibrinolytic system can lead to severe bleeding.^(9)^ Diagnosis of DIC is not simple; it depends on the association of compatible clinical signs and symptoms, which include laboratory clotting test results that can be altered in the presence of any disease that can lead to DIC. Many clinical conditions can compromise laboratory parameters obtained to diagnose DIC, including platelet count, prothrombin time (PT), activated prothrombin time (aPTT), fibrinogen concentration and fibrin degradation products (FDPs). Low levels of fibrinogen are associated with a poor outcome. For the diagnosis of DIC, many scores have been proposed.^(7)^ The current standard-of-care diagnostic strategy, the International Society on Thrombosis and Hemostasis (ISTH) scoring system, relies on results from 4 widely available assays.^(10)^ The standard parameters of the ISTH algorithm (platelet count, PT, fibrinogen, and D-dimer) are less useful individually than when considered jointly as a score capable of identifying the multifactorial disorders of hemostasis in DIC. Alternatively, the thromboelastometry score is designed to maximize the detection of the hypocoagulable state typical of patients in the advanced stages of DIC, including impairment of the enzymatic system (coagulation factors), cellular factors (thrombocytopenia) and the fibrin substrate (fibrinogen).^(10)^ Point-of-care testing for DIC is a highly desirable goal. To establish an early diagnosis, expected laboratory results associated with the clinical condition are necessary to guide therapy goals and predict prognosis. Given the dynamic characteristics of coagulopathies, repeated diagnostic tests are required to monitor the response to therapy to avoid inappropriate use of blood components or hemostatic drugs.

## CASE REPORT

**Figure 1:**
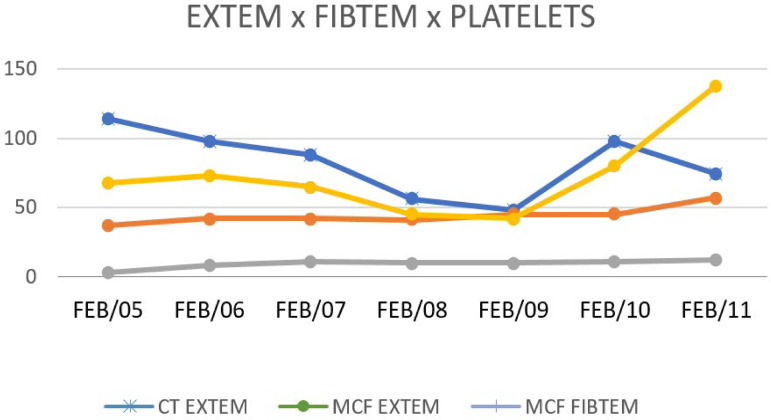
Comparison of thromboelastometry parameters and platelet count. EXTEM - extrinsic pathway; FIBTEM - cytochalasin D; CT - clot time; MCF - maximum clot firmness.

We report the case of a 23-year-old male admitted to the Department of Critical Care with sudden onset of fever, abdominal pain, prostration, generalized muscle pain associated with jaundice, liver failure, and DIC. The patient had no reported history of vaccination for yellow fever. He had been feeling sick, with generalized muscle pain and inappetence for the past 3 days. High fever, nausea, vomiting and diarrhea appeared within the previous 24 hours. No abdominal pain was reported. Diuresis was present, but the urine was concentrated, and there were no other symptoms. The only medication taken by the patient was dipyrone 500mg. He noted that he used to travel every week to a suburban city called Atibaia. The physical examination showed a good general state but severe dehydration with fever. The patient was awake without clinical signs suggestive of meningeal injury. Respiratory and cardiac examinations were normal. The following results were found: blood pressure, 135 x 80mmHg; heart rate, 91bpm; and axillary temperature, 39°C. His skin appeared normal. Laboratory tests showed the following results: leukocytes, 2.830/mm^3^; lymphocytes, 962/µL; neutrophils 1,075/µL; platelets 65.000/mm^3^; hemoglobin 15g/dL; hematocrit, 44%; PT activity, 85%; international nationalized ratio, 1.09; aPTT, 36 seconds; fibrinogen, 188mg/dL; aspartate aminotransferase (AST), 1,650U/L; alanine aminotransferase (ALT), 892U/L; total bilirubin, 0.8mg/dL; and C-reactive protein, 2.2mg/dL. There was no metabolic acidosis. Serology and rapid tests for dengue virus were negative. Since conventional laboratory tests showed only thrombocytopenia, thromboelastometry was required to confirm the presence of coagulopathy, and the following results were obtained: clot time (CT) in extrinsic pathway (EXTEM) of 88 seconds; clot formation time (CFT) in EXTEM of 194 seconds; alpha angle was 57º; maximum clot firmness (MCF) in EXTEM of 42mm; maximum lysis (ML) was 13%; and MCF cytochalasin D (FIBTEM) was 9mm. ROTEM® showed a hypocoagulable state based on impairment in clot strength (MCF EXTEM), with slight hypofibrinogenemia based on the results within the lower limit of the normal range for the MCF FIBTEM value. The clinical picture suggested a diagnosis of liver failure (Figure 1). Disseminated intravascular coagulation was confirmed by the ISTH score. Coagulopathy was monitored by thromboelastometry, and hemostatic therapy was initiated. Fibrinogen concentrates and vitamin K were given to avoid spontaneous bleeding.

## DISCUSSION

This case report illustrates the role of thromboelastometry as a tool to identify coagulation disorders associated with liver failure and DIC due to yellow fever. In this case, conventional laboratory tests showed thrombocytopenia and normal levels of fibrinogen (based on the Clauss assay). In the traditional therapeutic strategy, blood component transfusion can be used, including platelet concentrates, fresh frozen plasma and cryoprecipitates, given the risk of life-threatening bleeding. Sarani et al. showed that fresh frozen plasma is associated with an increased risk of nosocomial infection.^(11)^ Khan et al. demonstrated that transfusion of any blood component was associated with an increased risk of acute pulmonary failure in critically ill patients, mainly with regard to platelet concentrates.^(12)^ Taylor et al. published in 2006 that red blood cell (RBC) transfusion is an independent risk factor for nosocomial infection, with mortality increasing from 10.2% to 21.8%.^(13)^

In this case report, sequential thromboelastometry analysis was performed due to the dynamic and complex coagulation disorder found in association with DIC symptoms. Thromboelastometry identified a hypocoagulable state within the patient with slight coagulation factor deficiency and fibrinogen consumption, and these results were used to guide the administration of vitamin K and fibrinogen concentrates to correct the coagulopathy. The reduction in MCF amplitude in the EXTEM test associated with the reduction in MCF amplitude in FIBTEM test suggested a high risk of spontaneous bleeding, which could occur in both the gastrointestinal mucosa and central nervous system.^(14)^ As the coagulopathy was due to the thrombocytopenia and low fibrinogen concentration, fibrinogen concentrate was administered to improve clot strength, removing the need for platelet concentrate transfusion. Studies in patients with trauma-induced coagulopathy and thrombocytopenia have demonstrated the role of fibrinogen concentrates in correcting coagulation disorders, without the need for transfusion of platelet concentrate.^(15)^

Current views on the physiology of the coagulation system have demonstrated the importance of the cell membrane in the process of thrombin generation and clot formation. Hoffman and Monroe in 2001^(16)^ showed that the so-called coagulation initiation and amplification phases, composed of extrinsic and intrinsic coagulation factors, act to generate thrombin. Once the thrombin burst has occurred, an unstable fibrin clot is formed in its substrate: fibrinogen in the propagation phase. Finally, there is stabilization of the fibrin clot as a result of the action of factor XIII, which binds the fibrin monomers and allows further downstream actions of the fibrinolytic system.

Inappropriate assessment of abnormal coagulation can lead to failure to correct the coagulopathy, leading to poor outcomes. Wide application of blood component transfusion can increase the risk of adverse events. In this case, administration of fibrinogen concentrates, and vitamin K guided by sequential thromboelastometry resulted in an effective correction of the coagulopathy (here, associated with DIC and liver failure) in a patient presenting with severe yellow fever.

While coagulation factors are involved in the thrombin generation process, factor XIII and the fibrinolytic system act in clot stabilization. Both fibrinogen and platelets have been considered determinants of clot strength. Thus, as demonstrated in this case, the use of vitamin K associated with the administration of fibrinogen concentrate increased thrombin generation and improved clot strength even in the presence of thrombocytopenia once the fibrinogen concentration had increased in the blood. No blood component transfusion was performed, and no other bleeding complications were identified.

Written informed consent was obtained from the patient for publication of this case report and any accompanying images.

## CONCLUSION

Thromboelastometry allowed the contemporary diagnosis of a complex and dynamic coagulopathy associated with yellow fever, guiding hemostatic therapy and enabling monitoring clinical response. The continuous administration of fibrinogen concentrates and vitamin K to correct the coagulation disorder prevented life-threatening hemorrhage-related complications until resolution of the infectious disease. Fibrinogen concentrates improved clot strength even in the presence of thrombocytopenia. No blood component transfusion was required.
